# Complete mitochondrial genome of *Sporophila maximiliani* (Ave, Passeriformes)

**DOI:** 10.1080/23802359.2017.1347840

**Published:** 2017-07-14

**Authors:** Sandra Ludwig, Ana Paula Vimieiro Martins, Ana Luiza Lemos Queiroz, Anderson Oliveira do Carmo, Bárbara Bruna Ribeiro Oliveira-Mendes, Evanguedes Kalapothakis

**Affiliations:** a Departamento de Zoologia, Instituto de Ciências Biológicas, Universidade Federal de Minas Gerais, Belo Horizonte, MG, Brasil;; b Laboratório de Biotecnologia e Marcadores Moleculares, Pós-Graduação em Genética, Instituto de Ciências Biológicas, Universidade Federal de Minas Gerais, Belo Horizonte, Brasil

**Keywords:** Bicudo, NGS, genome, wildlife traffic, birds, mtDNA

## Abstract

*Sporophila maximiliani*, commonly known as Great-billed Seed-Finch or ‘bicudo’, is a trafficked bird in Brazil due to the species’ beauty and singing, which is appreciated by breeders and collectors. Generally, the Environmental Military Police and IBAMA maintain enforcement actions, rescue work, and seizure of illegally traded of ‘bicudo’ specimens. The genomic DNA of one specimen was sequenced on MiSeq (Illumina) sequencer. The reads obtained were analyzed, trimmed, and *de novo* assembled using CLC Workbench^®^ v9.0 (CLC Bio-Qiagen). The mitochondrial genome of *S. maximiliani* consisted of 16,765 base pairs, 2 ribosomal RNA, 22 transporter RNA, 13 protein-coding genes, and 1 control region. The molecular phylogeny demonstrated that the mitochondrial genome of *S. maximiliani* diverged from others related Passeriformes.

## Introduction

Estimates indicate ∼38 million wildlife animals are trafficked from Brazil annually and illegally commercialized (Destro et al. 2012). The National Network for Combating Wild Animal Trafficking of Brazil, which include Military Police and the Brazilian Environmental Agency (IBAMA), determined 86% of the bird species (Renctas, 2001). A high Passeriformes prevalence is observed in trafficking, which can be explained primarily by many Brazilians desire to keep members of this order of birds in cages based on an appreciation of their beauty and singing. According to Destro et al. (2012), *Sporophila maximiliani* possesses these attributes and is therefore a desired species by collectors and breeders, which correspond to 9th place in the ranking of trafficked the birds in Brazil. However, the proper morphological identification, during trafficked animal rescue efforts, is a difficult task at the species level because of the sexual dimorphism or even more complicated because they are confounded with juveniles of yours counterparts. In this way, the objective of this study was to obtain the mitochondrial genome of *S. maximiliani* and examine the phylogenetic relationship with four other closely related Passeriformes species, that could improve the techniques of species identification.

**Figure 1.**
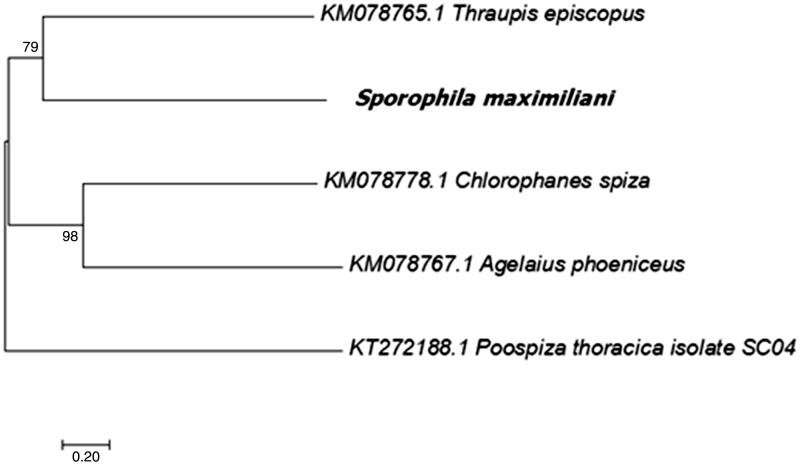
Molecular phylogeny of *Sporophila maximiliani* mitochondrial genome, based on the maximum likelihood (ML) method using a Kimura 2-parameter model (Kimura 1980). The bootstrap values are shown at node branches (>50). Initial tree(s) for the heuristic search were obtained by applying the Neighbor-Joining method to a matrix of pairwise distances estimated using the Maximum Composite Likelihood (MCL) approach. A discrete Gamma distribution was used to model evolutionary rate differences among sites (5-categories (+*G*, parameter = 200.0000)). The rate variation model allowed for some sites to be evolutionarily invariable ([+*I*], 0.3734% sites). The tree is drawn to scale, with branch lengths measured in the number of substitutions per site. The analysis involved five mitochondrial genome sequences (see details above).

*Sporophila maximiliani* blood samples were provided by IBAMA (with proper licensing from the competent authorities: SISBIO:56471-1, IEF:024/2016 and CEUA-UFMG 37/2017) and genomic DNA was extracted following the phenol-chloroform protocol of Sambrook and Russell (2006), which was analyzed for purity, concentration and quality using spectrophotometry (NanoDrop), fluorometry (Qubit 2.0) and 0.7% agarose gel electrophoresis, respectively. Blood samples were stored at the Taxonomic Collection Center of the Universidade Federal de Minas Gerais (deposit code: UFMG FD 170023). Subsequently, the genomic library was constructed using 50 ng of DNA following the Nextera DNA Library Preparation Kit (Illumina Inc., San Diego, CA) and sequenced using the MiSeq^®^ Reagent Kit V3-600 (Illumina) with paired-end strategy. The obtained reads were analyzed based on quality using FASTQC (available at http://www.bioinformatics.babraham.ac.uk/projects/fastqc/). CLC Workbench^®^ v9.0 (CLC Bio-Qiagen) software was employed to trimmed and *de novo* assembly reads. Approximately 21,669 paired sequence reads were used to produce the complete mitochondrial DNA (mtDNA) genome with average coverage of 72×. The annotation of the obtained genome was performed at MITOS Web Server (Bernt et al. 2013). Subsequently, the mtDNA genome was submitted to Standalone BLAST (Tao 2016), which resulted in similarity with other Passeriformes species and, from those, four mitochondrial genome were used against *S. maximiliani* mtDNA genome: *Thraupis episcopus* Linnaeus 1766*, Chlorophanes spiza* Linnaeus 1758*, Agelaius phoeniceus* Linnaeus 1766, and *Poospiza thoracica* Nordmann 1835 (GenBank accession numbers: KM078765; KM078778; KM078767; KT272188, respectively). The obtained sequences were aligned using MEGA 7.0.21 (Kumar et al. 2016) and the best substitution model was evaluated by JModelTest (Posada 2008). Subsequently, using MEGA, the molecular phylogenetics from *S. maximiliani* was inferred (Figure 1).

The complete *S. maximiliani* mtDNA consisted of 16,765 bp and is available at GenBank (Accession no. MF327582). Results indicated the GC content of paired sequences was 42%; base frequencies were 24.3% A, 32% G, 28.5% T, and 15% C. The mtDNA structure contained 2 rRNA (rrnS and rrnL), 22 tRNA (trnE, trnF, trnV, trnL2, trnI, trnQ, trnM, trnW, trnA, trnN, trnC, trnY, trnS2, trnD, trnK, trnG, trnR, trnH, trnS1, trnL1, trnT and trnP), 13 protein-coding genes (nad1, nad2, cox1, cox2, atp8, atp6, cox3, nad3, nad4l, nad4, nad5, cob and nad6) and 1 control region (dloop).
